# High Profile Transvalvular Pump Assisted Recovery for Takotsubo Cardiomyopathy: A Case Series

**DOI:** 10.3390/jcm14093225

**Published:** 2025-05-06

**Authors:** Jordan Young, Patrick McGrade, Jaime Hernandez-Montfort, Jerry Fan

**Affiliations:** Department of Internal Medicine, Baylor Scott and White Health—Temple, Temple, TX 76508, USA; jordan.young1@bswhealth.org (J.Y.); patrick.mcgrade@bswhealth.org (P.M.); jaime.hernandezmontfort@bswhealth.org (J.H.-M.)

**Keywords:** high profile transvalvular pump, mechanical circulatory support, stress-induced cardiomyopathy

## Abstract

**Background:** Stress-induced cardiomyopathy (SI-CM) is a transient left ventricular dysfunction triggered by emotional or physical stress, often resolving with supportive care. However, severe cases may progress to cardiogenic shock (CS), requiring mechanical circulatory support (MCS). High-profile transvalvular pumps (HPTP), a form of percutaneous ventricular assist device, offer promising hemodynamic support in acute heart failure. This report explores HPTP use in SI-CM-related CS through two complex clinical cases. **Case Summary:** Two elderly female patients presented with severe CS secondary to apical-variant SI-CM. Case 1 involved a 67-year-old woman with sepsis, colonic perforation, and recurrent SI-CM, leading to profound low-output shock despite multiple vasopressors and inotropes. HPTP was implanted via the axillary artery, allowing for surgical management of intra-abdominal pathology and eventual cardiac recovery. Case 2 featured a 77-year-old woman with multifocal pneumonia, severe mitral regurgitation, and complete heart block. HPTP implantation stabilized her hemodynamics, facilitated extubation, and led to full recovery of ventricular function. **Results:** Both patients showed marked improvement in cardiac output and systemic perfusion following HPTP insertion. Echocardiograms post-device removal revealed normalization of left ventricular ejection fraction (55–64%). Hemodynamic data confirmed reduced pulmonary capillary wedge pressure and systemic vascular resistance. **Conclusion:** These cases highlight the potential of HPTP in managing SI-CM-related CS, especially when traditional therapies are inadequate or contraindicated. HPTP can rapidly restore hemodynamic stability and support myocardial recovery. While current data are limited, these observations underscore the need for broader investigation into the role of HPTP in this setting.

## 1. Introduction

Stress-induced cardiomyopathy (SI-CM), is a reversible condition characterized by a sudden weakening of the heart muscle, often triggered by emotional or physical stress. While most patients with SI-CM recover with supportive care and medical treatment, severe cases may require temporary mechanical circulatory support (MCS) to assist the heart during the recovery phase. High profile transvalvular pumps (HPTP) are percutaneous ventricular assist device that has shown promise in providing hemodynamic support for patients with various forms of heart failure. This paper aims to explore the role of HPTP-assisted recovery in SI-CM, examining its benefits, challenges, and outcomes in this unique clinical scenario.

This paper details two cases of patients who were admitted to our facility with SI-CM and later progressed to severe cardiogenic shock (CS), necessitating the use of a HPTP device for MCS.

## 2. Case Description

### 2.1. Case 1

A 67-year-old woman with hypertension, hyperlipidemia, and ischemic cardiomyopathy was admitted to the hospital as a trauma activation following a fall from ground level. The fall was triggered by pre-syncopal symptoms. Before the fall incident, she experienced two months of bloody diarrhea and an unintentional weight loss of 40 pounds. She presented with low blood pressure (70 s/40 s mmHg), a significant increase in white blood cell count (leukocytosis of 18.2 × 10*9/L, normal range: 4.8–10.8 × 10*9/L), lactic acidosis (4.3 mmol/L, normal range: 0.5–2.0 mmol/L), elevated B-type natriuretic peptide (>5000 pg/mL, normal range: 0–100 pg/mL), and elevated cardiac enzymes (troponin I 0.70 ng/mL, normal range: 0.00–0.09 ng/mL). A computed tomography of the abdomen and pelvis revealed marked irregular wall thickening in the rectosigmoid colon, suggesting a possible malignancy, as well as pneumoperitoneum.

**Figure 1:**
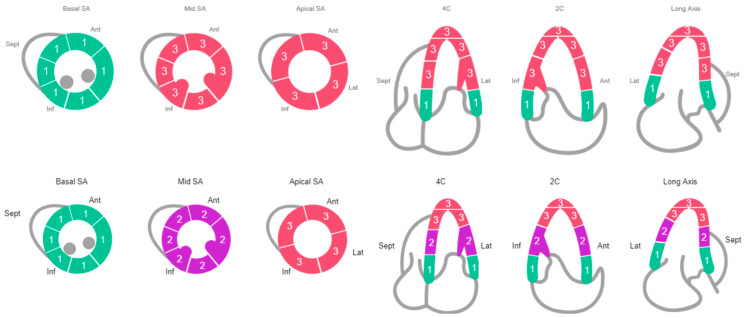
Echocardiogram: Row 1 (Case #1 Schematic of Regional Wall Motion), Row 2 (Case #2 Schematic of Regional Wall Motion).

**Figure 2:**
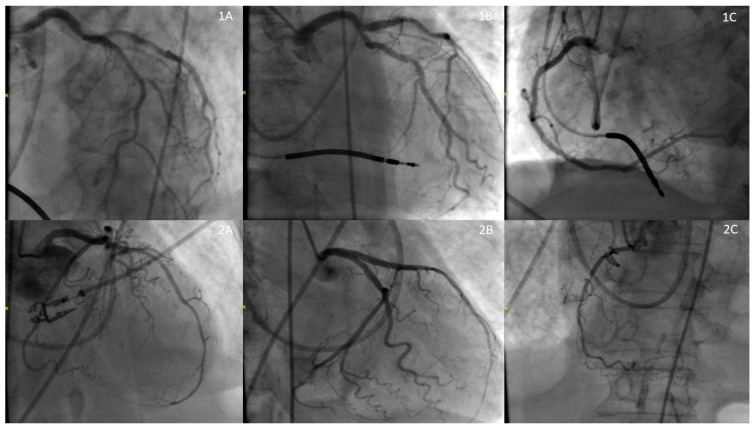
Coronary Angiogram: Row 1 (**1A**: Left Anterior Descending Artery (50%), **1B**: Left Circumflex Artery (50%), **1C**: Right Coronary Artery (0%)), Row 2 (**1A**: Left Anterior Descending Artery (50%), **1B**: Left Circumflex Artery (0%), **1C**: Right Coronary Artery (90%)).

She was immediately treated with pressors and started on empiric antibiotics (vancomycin and piperacillin-tazobactam). An echocardiogram showed a severely reduced ejection fraction of <20% with regional wall motion abnormalities consistent with SI-CM (apical akinesis with hyperdynamic basal segments), despite her previous recovery from cardiomyopathy (Figure 1). A coronary angiogram was performed, which indicated non-obstructive coronary artery disease (Figure 2).

About 5 years prior to her current presentation, she had been hospitalized for acute decompensated heart failure. At that time, an echocardiogram revealed a greatly enlarged left ventricle with an ejection fraction of 10–15% and regional wall motion abnormalities, all attributed to coronary artery disease. She underwent percutaneous coronary intervention as she was not a suitable candidate for coronary artery bypass grafting. Despite three months of guideline-directed medical therapy, her ejection fraction did not improve sufficiently, leading to the implantation of a single-chamber implantable cardioverter defibrillator. During the subsequent four years, she was lost to follow-up, but upon reevaluation, her echocardiogram showed an improved ejection fraction of 52% without any regional wall motion abnormalities.

**Figure 3:**
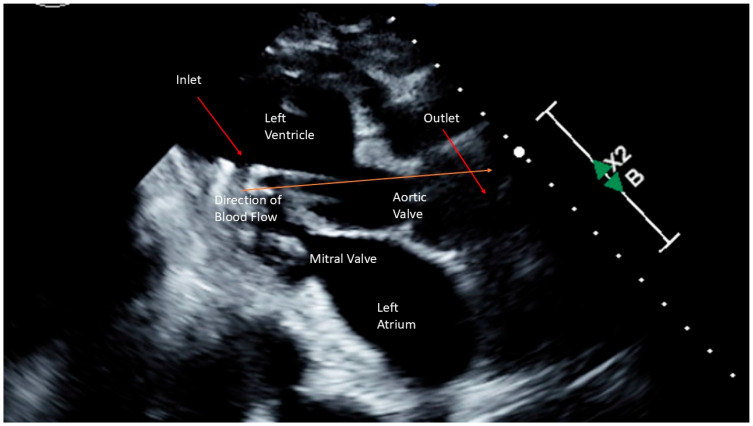
Schematic Representation of a High Profile Transvalvular Pump.

Despite efforts to increase her heart’s pumping ability using inotropes and pressor support, she remained in a low-output state. (Table 1) Consequently, HPTP was surgically implanted via the right axillary artery. (Figure 3) After implantation, she was provided 4.5 L/min of support (maximal support for a HPTP is 5.5 L/min). Concurrently, colorectal surgery performed an exploratory laparotomy, sigmoidectomy, and small bowel resection. The anatomical pathology results indicated diverticular disease with abscess formation, and peritoneal fluid culture identified extended spectrum beta-lactamase Escherichia coli as the causative agent. Her antibiotics were switched to a 14-day course of levofloxacin, targeted to treat the infection.

After a month of recovery, the HPTP device was successfully removed, and a subsequent echocardiogram revealed an improved ejection fraction of 55–60%. She was discharged on a combination of aspirin, atorvastatin, clopidogrel, dapagliflozin, furosemide, lisinopril, metoprolol, and spironolactone.

### 2.2. Case 2

A 77-year-old female with migraine headaches, osteoarthritis, hypothyroidism, hypercholesterolemia, and depression presented to the Emergency Department with several days of progressively worsening shortness of breath, nonproductive cough, and nausea. She denied chest pain, and review of systems was otherwise negative. Four days prior to presentation, she underwent dental implant surgery. Prior to symptom onset, the patient had been in her usual state of health, remaining active, living independently, and performing all activities of daily living without assistance.

Her vital signs were notable for tachycardia (100–110 beats per minute), tachypnea (23–25 breaths per minute), and blood pressure on the lower end of normal (90–110 s/70–90 mmHg). Laboratory results showed lactic acidosis (4.0 mmol/L, normal range: 0.5–2.0 mmol/L), elevated white blood cell count (11.5 × 10*9/L, normal range: 4.8–10.8 × 10*9/L), elevated B-type natriuretic peptide (699 pg/mL, normal range: 0–100 pg/mL), and elevated cardiac enzymes (troponin I 1.81 ng/mL, normal range: 0.00–0.09 ng/mL). Electrocardiogram showed sinus tachycardia with lateral T wave abnormalities but no ST elevations. Computed tomography pulmonary angiography ruled out pulmonary embolism, revealing instead multifocal pneumonia and bilateral pleural effusions. She was intubated due to severe respiratory distress and admitted to the intensive care unit.

Broad-spectrum antibiotics were initiated for the infection. An echocardiogram revealed an ejection fraction of 34% with regional wall motion consistent with SI-CM with functional severe mitral regurgitation (Carpentier’s Type IIIb due to restricted leaflet closure) (Figure 1). Coronary angiography showed non-obstructive coronary artery disease. (Figure 2) A right heart catheterization was performed, revealing SCAI Stage D CS with a cardiac output of 1.65 L/min (normal range: 4–8 L/min), a cardiac index of 1.19 L/min/m^2^ (normal range: 2.5–4 L/min/m^2^), and severely elevated systemic vascular resistance of 4500 dynes s/cm^5^ (normal range: 800 and 1200 dynes s/cm^5^). The patient also had severe pre- and post-capillary pulmonary hypertension, with a pulmonary vascular resistance of 4.84 Wood units (normal <2 Wood units) and a pulmonary capillary wedge pressure of 31 mmHg (normal range: 4–12 mmHg). Additionally, the patient developed complete heart block during the catheterization requiring placement of a temporary screw-in pacemaker.

Despite aggressive management, the patient’s condition continued to deteriorate, with increasing requirements for vasopressors and inotropes, worsening lactic acidosis, and reduced urine output. (Table 1) The decision was made to place a HPTP for enhanced support. (Figure 3) After implantation, she was provided 3.5 L/min of support (maximal support for a HPTP is 5.5 L/min). The patient had subsequent improvement in hemodynamics and was extubated the following morning. After 8 days of recovery, the HPTP device was removed. A repeat echocardiogram demonstrated complete recovery with ejection fraction of 64%. She was discharged on a combination of aspirin, furosemide, hydralazine, and metoprolol.

**Table 1:** Hemodynamics.

Characteristic	Patient 1	Patient 2
Age/Sex	67/Female	77/Female
LVOTO	No	No
Comorbid Conditions	Septic Shock, Small Bowel Obstruction, Colonic Perforation	Severe Mitral Regurgitation
Mechanical Ventilation	Yes	Yes
Hemodynamics		
Before HPTP		
AO (mmHg)	63/45 (54)	95/71 (79)
PA (mmHg)	45/32 (38)	49/32 (39)
PCWP (mmHg)	31	31
CI (L/min/m^2^)	1.8	1.2
SVR (dynes*s/cm^5^)	1175	4500
Type and Dose of Inotrope/Vasopressor	Dobutamine 10 mcg/kg/min Epinephrine 0.3 mcg/kg/min Norepinephrine 0.3 mcg/kg/min Vasopressin 0.06 mcg/kg/min Milrinone 0.375 mcg/kg/min Phenylephrine 180 mcg/min	Dobutamine 5 mcg/kg/min Epinephrine 0.08 mcg/kg/min Norepinephrine 0.13 mcg/kg/min
After HPTP		
AO (mmHg)	88/70 (75)	108/65 (79)
PA (mmHg)	30/16 (23)	35/15 (22)
PCWP (mmHg)	5	6
CI (L/min/m^2^)	2.7	2.5
SVR (dynes*s/cm^5^)	1588	1477
Type and Dose of Inotrope/Vasopressor	24 h After Placement Dobutamine 5 mcg/kg/min Epinephrine 0.04 mcg/kg/min Angiotensin II 8 ng/kg/min	24 h After Placement Dobutamine 5 mcg/kg/min

Note: Tablet Abbreviations: LVOTO: Left Ventricular Outflow Tract Obstruction, AO: Aortic Pressure, PA: Pulmonary Artery Pressure, PCWP: Pulmonary Capillary Wedge Pressure, CI: Cardiac Index, SVR: Systemic Vascular Resistance. For AO/PA: reported as systolic/diastolic (mean). PCWP: reported as mean.

## 3. Discussion

HPTP are temporary MCS device that have a unique ability to unload the left ventricle and improve coronary perfusion while reducing myocardial workload [1]. The use of HPTP in SI-CM patients with CS remains poorly studied. The device’s capability to deliver immediate hemodynamic support has been linked to better outcomes in high-risk patients, including those undergoing high-risk percutaneous coronary intervention or experiencing CS due to acute myocardial infarction or cardiomyopathy (such as peripartum cardiomyopathy or myocarditis) [2]. A HPTP can be deployed quickly, enabling timely intervention in critical situations and acting as a bridge to recovery or definitive therapies.

SI-CM is defined as transient regional left ventricular systolic dysfunction in the absence of obstructive coronary artery disease or plaque rupture [3]. SI-CM is typically associated with a physical, emotional, or psychological stressor [3]. There are several variants of SI-CM including apical (most common), mid-ventricular, basal, and focal (least common) [3,4]. In both of our cases, they had the most common apical variant which is characterized by dilation and akinesis of the left ventricular apical wall segments, often described as “apical ballooning”, along with hyperkinesis of the left ventricular basal wall segments which may lead to left ventricular outflow obstruction [3]. SI-CM is a diagnosis of exclusion; however, echocardiography can be helpful in the diagnosis because the regional wall motion abnormalities usually extend beyond a single coronary distribution [3].

The exact pathophysiology of SI-CM remains unclear, though the association with stressors suggest that excessive catecholamine release or activation leads to myocardial toxicity, coronary vasospasm, and microvascular dysfunction, resulting in myocardial injury and transient left ventricular dysfunction [5,6]. The transient nature of SI-CM results in a wide spectrum disease severity at diagnosis, ranging from asymptomatic to fulminant CS or even sudden cardiac death [5]. The management of SI-CM ranges from supportive care to invasive therapies and often necessitates intensive care unit admission [3]. While treatment strategies are generally based on principles used for conditions like heart failure, left ventricular outflow tract obstruction, and dysrhythmias, it is essential to consider the distinct pathophysiology of SI-CM, as this can help guide therapeutic decisions [5].

CS is a serious complication of SI-CM, with reported incidence between 5 to 20% [3,7]. Patients with SI-CM who develop CS have significantly worse outcomes. In one comparative study that examined 260,144 cases of SI-CM, in-hospital mortality was estimated to be 6-fold higher in those who developed CS compared with those without CS (23% vs 4%, *p* < 0.01) [8]. Independent predictors of mortality included male gender and increased burden of pre-existing comorbidities such as congestive heart failure, chronic pulmonary disease, and diabetes [8].

The use of vasopressors or inotropic agents remains controversial in CS due to the excessive catecholamines as a driving force for worsening cardiomyopathy [9,10]. Some studies advocate for complete avoidance, citing the pathophysiological role of catecholamine excess, making inotropes and vasopressors potentially ineffective or even harmful [9,10]. In clinical practice, these agents are often initiated while investigation is ongoing, prior to the recognition of SI-CM, or as part of the immediate stabilization process.

MCS is frequently used in the management of SI-CM related CS and can help provide rapid hemodynamic stabilization especially if vasopressors or inotropic agents are contraindicated [11]. MCS may offer an alternative for SI-CM related CS, helping to avoid the complications or inconsistent responses seen with inotropes [5,11,12]. For these reasons, Madias et al. propose early initiation of MCS for patients with SI-CM related CS [5]. Data from the International Takotsubo Registry demonstrated that those suffering from SI-CM related CS who were treated with MCS had a lower in-hospital mortality (12.8%) compared to those who were not treated with MCS (28.3%) [13].

Several different types of MCS devices exist including intra-aortic balloon bump (IABP), extracorporeal membrane oxygenation (ECMO), CentriMag, TandemHeart, and Impella devices [2]. These devices serve to provide short-term support and unloading of the left ventricle during recovery or, in those who do not recovery a temporizing measure until more long-term therapies can be implemented [2]. The Impella is a HPTP continuous-flow axial pump situated across the aortic valve which helps to offload the heart by pulling blood from the left ventricle and redirecting flow into the aorta [1]. This hemodynamic support is achieved by increasing cardiac output, reducing myocardial oxygen consumption, reducing pulmonary capillary wedge pressure, and improving systemic perfusion [1].

HPTP were not approved for use in the United States until 2019, which may explain the limited literature describing its application. We found a single case report documenting its use in SI-CM related CS. [10] This report detailed a 77-year-old female who had recently undergone a minimally invasive surgical mitral valve replacement and subsequently developed acute heart failure secondary to SI-CM. She ultimately required MCS with an HPTP. Like the two patients in our study, her hemodynamics improved immediately after implantation, and similar to patient #2, she was extubated the following day. The HPTP was removed after five days, and by day ten, complete recovery of her ejection fraction to 55% [10].

Historically, IABP and veno-arterial ECMO have been more widely reported in the literature, largely due to their decades-long use in clinical practice [12]. More recently, HPTP devices have been introduced, and its use in SI-CM related CS is gaining traction. Over time, reports of ECMO and HPTP use have increased, while those of IABP have declined [11].

HPTP offers several advantages over traditional devices. Unlike IABP, which requires some degree of native left ventricular function, HPTP can provide direct left ventricular unloading [1]. Additionally, while IABP reduces left ventricular afterload, its placement in the descending aorta can exacerbate or reveal left ventricular outflow tract obstruction in SI-CM, potentially increasing pressure gradients and worsening hemodynamics. Moreover, IABP has limited cardiac index augmentation, which may be inadequate in severe CS cases [14].

On the other hand, veno-arterial ECMO offers full circulatory support but can increase left ventricular end-diastolic pressure and afterload, both of which are often already elevated in CS [14]. Compared to veno-arterial ECMO, HPTP are less invasive and has been associated with lower device-related complication rates [14,15]. Additionally, a HPTP may facilitate quicker left ventricular recovery. In a systematic review by von Mackensen et al. [16], which included 124 patients with SI-CM related CS, 50% received veno-arterial ECMO, 35.5% received HPTP, and 10.5% received IABP [16]. HPTP use was significantly associated with shorter time to cardiac recovery (*p* < 0.001) [16]. Since MCS-related complications increase with longer support duration, the HPTP ability to expedite left ventricular recovery may reduce overall morbidity [10].

SI-CM is part of a spectrum of transient left ventricular dysfunction, a reversible cause of cardiomyopathy [17]. The etiology is diverse including stress (physical, emotional, or psychological), neurogenic (central nervous system injuries), allergic reactions, myocarditis, pregnancy, and non-obstructive coronary artery disease making both diagnosis and treatment challenging [17]. Due to multiple distinct pathophysiological mechanisms it is important to tailor your clinical, imaging, and biomarker assessment to quickly elucidate the underlying cause which will help tailor therapy [17]. However, common underlying factors often help predict left ventricular recovery including the lack of late gadolinium enhancement on cardiac magnetic resonance imaging, low or absent troponin and B-type natriuretic peptide and higher left ventricular systolic function at diagnosis are all predictors of a shorter recovery time and better prognosis [17].

## 4. Conclusions

A HPTP provides full circulatory support and may shorten the time to left ventricular recovery. Its ability to reduce afterload and left ventricular end-diastolic pressure without exacerbating left ventricular outflow tract obstruction makes it an attractive option for SI-CM related CS, potentially leading to shorter intensive care unit stays and faster patient recovery. While data on HPTP use in SI-CM remains limited, case reports and small studies suggest promising outcomes. Patients receiving HPTP support often show rapid hemodynamic improvement and may experience faster and more complete recovery of left ventricular function. However, larger studies are needed to define the role of HPTP-assisted recovery in SI-CM more conclusively.

## Data Availability

The original contributions presented in this study are included in the article. Further inquiries can be directed to the corresponding author.
